# Insall–Salvati Ratio and Femoral Cartilage Thickness: Ultrasonographic and Clinical Evaluation in Knee Osteoarthritis

**DOI:** 10.5152/ArchRheumatol.2026.26308

**Published:** 2026-04-27

**Authors:** Nevzat Yesilmen, Muhammet Şahin Elbastı, Muhammed Korkmaz

**Affiliations:** Department of Physical Medicine and Rehabilitation, Elazığ Fethi Sekin City Hospital, Elazığ, Türkiye

**Keywords:** Femoral cartilage thickness, Insall**–**Salvati ratio, knee osteoarthritis, ultrasonography

## Abstract

**Background/Aims:**

This study aimed to evaluate the relationship between the Insall–Salvati ratio (ISR) and femoral cartilage thickness (FCT) in patients with knee osteoarthritis (KOA), and to examine their associations with radiographic disease stage, clinical findings, and functional capacity, including the ability of mean FCT to discriminate late-stage KOA.

**Materials and Methods:**

This cross-sectional study included 104 patients (52/group) diagnosed with KOA via American College of Rheumatology criteria: early stage (Kellgren–Lawrence [K–L] 1-2) and late stage (K–L 3-4). One index knee per participant was analyzed. The ISR was measured on radiographs, and FCT on ultrasonography. Visual Analog Scale (VAS), Knee Injury and Osteoarthritis Outcome Score (KOOS), Western Ontario and McMaster Universities Osteoarthritis Index (WOMAC), and 100-m walking time were recorded. Data were analyzed using age- and body mass index (BMI)-adjusted ANCOVA. Stage-specific Pearson correlations were adjusted via the Benjamini–Hochberg false discovery rate (FDR) procedure. Multivariable regression and receiver-operating characteristic curve analyses were also conducted.

**Results:**

After FDR correction, mean ISR correlated with FCT in late-stage KOA (*r* = 0.527, q < 0.001) but not in early-stage KOA (*r* = 0.331, q = 0.058). Age- and BMI-adjusted ANCOVA confirmed between-stage differences in mean FCT, VAS, KOOS, WOMAC, and 100-m walking time (all *P* < .001); however, mean ISR did not differ by stage (*P* = .419). In multivariable regression, late-stage KOA (*B* = −0.411 mm) and mean ISR (*B* = 0.829 mm per 1-unit increase) were independently associated with mean FCT (*R*^2 ^= 0.463). Mean FCT discriminated late-stage KOA (area under the curve = 0.845, 95% CI: 0.771-0.919); a cutoff of ≤1.82 mm yielded 63.5% sensitivity and 88.5% specificity.

**Conclusion:**

Ultrasonographic FCT is strongly associated with radiographic stage, pain, functional scores, and walking performance, showing good discrimination of late-stage KOA. While ISR was not stage discriminative, it was independently associated with FCT. These findings should be viewed as exploratory; causal inferences cannot be drawn from this cross-sectional design.

**Main Points**

- Ultrasonographic femoral cartilage thickness (FCT) differentiates early- and late-stage knee osteoarthritis (KOA) and is consistently associated with pain (Visual Analog Scale), functional scores (Knee Injury and Osteoarthritis Outcome Score/Western Ontario and McMaster Universities Osteoarthritis Index), and 100-m walking time, even after adjustment for age and body mass index.
- Mean Insall–Salvati ratio (ISR) does not independently differentiate radiographic stages after adjustment (*P* = .419), supporting a limited role of ISR as a stand-alone marker of KOA severity.
- After Benjamini–Hochberg false discovery rate correction, the ISR–FCT correlation remained robust in late-stage KOA (*r* = 0.527, q < 0.001) but did not reach statistical significance in early-stage KOA (*r* = 0.331, q = 0.058).
- Multivariable regression indicated that KOA stage and ISR were independently associated with mean FCT (*R*^2 ^= 0.463), and receiver-operating characteristic curve analysis showed good discrimination of late-stage KOA by mean FCT (area under the curve = 0.845); the ≤1.82 mm cutoff was internally derived and requires external validation.

## Introduction

Osteoarthritis (OA) is a chronic, progressive degenerative joint disease characterized by the gradual deterioration of articular cartilage and structural changes in surrounding tissues.^1^ Knee osteoarthritis (KOA) is one of the most common and functionally burdensome forms of the disease, affecting approximately 10% of men and 13% of women over 60 years of age.^2^ Although cartilage degeneration involves complex inflammatory and biochemical pathways,^3^ mechanical loading and malalignment are key contributors to OA development and structural deterioration.^2^^,^^3^ In particular, within the patellofemoral (PF) compartment, patellar malposition may increase focal contact stresses on articular cartilage and potentially accelerate degenerative changes.^4^^,^^5^

One of the structural factors known to affect PF alignment is patellar height. This anatomical feature plays a role in the distribution of loads on the knee joint, contributing to various clinical outcomes related to cartilage health.^4^^,^^5^ The Insall–Salvati ratio (ISR), frequently used in the assessment of patellar height, is defined as the ratio of patellar tendon length (PTL) to diagonal length of the patella (DLP). This measurement can be reliably performed on both lateral knee radiographs and sagittal magnetic resonance imaging (MRI) sections.^6^ The ISR is considered one of the most frequently used methods for detecting height abnormalities such as patella alta and patella baja due to its high intra-observer and inter-observer reliability.^7^

The role of ISR in KOA has mostly been discussed in the literature through the PF joint component. However, evidence regarding its association with the tibiofemoral (TF) compartment is also increasing. For instance, a recent cross-sectional study by Yang et al^^8^^ reported that a higher Insall–Salvati–based patellar height ratio is positively associated with overall KOA severity, while other PF alignment parameters show strong correlations with the radiographic severity of TF OA. Taken together, these findings suggest that abnormal patellar height and malalignment may be associated with the structural (radiographic) severity of the TF compartment, although causality cannot be inferred from this study design.^8^ On the other hand, in the study by Doruk Analan et al,^^9^^ no significant relationship was found between ISR and symptomatic measures such as pain, functional capacity, and postural balance. This finding suggests that the prognostic value of ISR in terms of clinical symptoms may be limited.^9^ The effect of patella height on knee biomechanics has been investigated in various studies. However, the knee joint is not a structure in which the PF and TF compartments work in isolation from each other in terms of biomechanics; on the contrary, it is a complex system in which these 2 compartments directly influence each other and form an integrated kinetic chain.^10^ The patella plays a key role in the transmission of force generated by the quadriceps muscle to the tibia as a fulcrum of the extensor mechanism. Changes in patellar height affect knee extension biomechanics by modifying the attachment angle of the patellar tendon to the tibia and the effective moment arm of the quadriceps muscle.^11^ This biomechanical change has the potential to alter not only the contact pressures in the PF joint but also the load transmission vectors and joint reaction forces in the TF compartment.^12^ Indeed, current radiological studies have shown that variations in patellar morphology and height are significantly associated with the severity of cartilage degeneration in the TF compartment.^13^ These findings suggest that patella height indices such as the ISR should be considered an important parameter not only in PF pathologies but also in the biomechanical and structural evaluation of TF OA.

Magnetic resonance imaging is considered the gold standard method for evaluating structural changes and early degeneration in TF articular cartilage. However, the high cost, long imaging time, and limited accessibility of MRI limit its applicability in routine clinical screenings. In contrast, musculoskeletal ultrasonography (US) stands out as a valid, reliable, non-invasive, and cost-effective alternative for evaluating femoral cartilage thickness (FCT).^14^^,^^15^ In fact, US has been shown to detect early-stage cartilage damage more accurately than conventional radiography and to correlate highly with histopathological findings.^16^ Accordingly, US can be considered a suitable and effective clinical imaging tool to examine the possible association between ISR and femoral cartilage (FC).

Although there are studies in the current literature that examine the relationship between ISR and radiographic OA staging or clinical symptoms, studies investigating the effect of patellar height (ISR) on “FCT,” which is a direct reflection of biomechanical effects, using ultrasonographic methods are quite limited. In this context, the main hypothesis of this study is that variations in patellar height (particularly low ISR values) are associated with a decrease in FCT, potentially through altered TF load distribution. Given the cross-sectional design and potential confounding, this hypothesis is evaluated as an exploratory association and is not interpreted as proof of biomechanical causality or as establishing ISR as an independent marker of radiographic severity.

Accordingly, the primary aim of this study is to examine the relationship between the radiographically measured ISR and the ultrasonographically measured FCT in patients with KOA. The secondary aim of the study is to evaluate whether these parameters differ according to KOA stages (early vs. late) and to examine their relationship with clinical/functional outcomes.

## Materials and Methods

### Study Design and Participants

This cross-sectional observational study was conducted among individuals who presented to the Physical Medicine and Rehabilitation outpatient clinic of Elazığ Fethi Sekin City Hospital with complaints of knee pain and were diagnosed with KOA between June 2024 and November 2024. The KOA diagnosis was made according to the American College of Rheumatology criteria. Radiological evaluation was performed according to the Kellgren–Lawrence (K–L) classification; K–L stages 1-2 were classified as early-stage KOA, and stages 3-4 as late-stage KOA.^^17^^

The required sample size and power analysis for the study were calculated using the G*Power Ver. 3.1.9.4 program. Based on the comparison of mean FCT scores in the study of Pane et al,^^18^^ it was concluded that there should be at least 52 people in each group with an effect size of 0.71 and a true power of 0.95, with an error margin of *α* = 0.05 and a desired power (1-*β*) = 0.95. Although radiological and clinical evaluations included bilateral knees, only 1 knee from each patient (“index knee”) was included in the analysis to ensure data independence in statistical analyses. The index knee was defined as the knee with the more advanced radiological stage in the presence of bilateral KOA, and as the more symptomatic side if both knees were at the same stage. With this method, individual-based independent analysis was used. For transparency, right and left knee measurements were also summarized and compared as an exploratory, descriptive analysis; these findings were not considered primary and were interpreted cautiously.

### Exclusion Criteria

Individuals under 45 and over 70 years of age were excluded from the study. To reduce confounding effects, isolated PF OA cases were excluded based on clinical examination (predominant anterior knee pain, positive patellar grind test) and available radiographic images. Patients with patellar fracture, surgical history, or severe dysplasia were also excluded. Furthermore, individuals with systemic diseases that could affect bone and cartilage metabolism, or those with risk factors that could affect this metabolism (e.g., active smoking, diabetes mellitus, and rheumatoid arthritis), were excluded.

### Ethical Approval and Information

This study was approved by the Fırat University Non-Interventional Research Ethics Committee (Approval No: 2024/07-31, May 9, 2024). All participants were given detailed information about the purpose and implementation process of the study; their verbal and written consent was obtained. The study was conducted in accordance with the ethical principles and rules of the Helsinki Declaration.

### Clinical and Demographic Data

Age, gender, and body mass index (BMI) (kg/m^2^) were recorded for all participants.

### Functional Evaluation

All patients were administered the Western Ontario and McMaster Universities Osteoarthritis Index (WOMAC) and the Knee Injury and Osteoarthritis Outcome Score (KOOS) scales to assess knee function and quality of life.

- The WOMAC scale consists of 24 items and is divided into 3 subgroups: pain (5 items), stiffness (2 items), and physical function (17 items). High scores indicate increased symptom severity and loss of function.^19^ The Turkish validity and reliability study of the scale was conducted by Tüzün et al.^20^
- The KOOS consists of a total of 42 items and 5 subscales (symptoms, pain, daily living function, sports and leisure activities, quality of life). Each item is scored between 0 and 4; subscales are evaluated by normalizing them between 0 and 100.^21^^,^^22^ The Turkish validity and reliability study of the scale was conducted by Paker et al.^23^

Both scales were completed by the patient and took approximately 10 minutes to finish.

- Pain intensity was evaluated using a 10-cm horizontal Visual Analog Scale (VAS). The extremes of the scale were anchored with “no pain” (0) and “worst imaginable pain” (10). Patients indicated their current pain level by placing a mark on the line. To ensure measurement accuracy, the distance from the left anchor to the patient’s mark was initially measured in millimeters (0-100 mm) and subsequently converted to a 0-10 score (by dividing by 10) for statistical analysis and reporting. The VAS is a widely recognized and validated instrument for assessing pain severity in patients with KOA.^24^

### Functional Performance Measurement

Patients’ walking capacity was assessed using the 100-meter walking time test (in seconds). This test has been described as a functional measurement tool with high test-retest reliability and validity used in clinical studies to assess walking performance.^25^ The test was performed on a flat and unobstructed surface, unsupported, and at the patient’s natural walking speed.

### Radiological Evaluation

Bilateral knee radiographs (anteroposterior and lateral) taken within the last 3 months and available in the hospital archive were reviewed. All radiographs were obtained in a standing weight-bearing position according to the institutional protocol. The anteroposterior view was acquired with the knee in extension, and the lateral view was acquired at approximately 30° knee flexion. The radiographs were evaluated via the picture archiving and communication system (PACS). The KOA staging was performed only according to the K–L classification. According to this classification, patients in stages 1-2 were included in the early-stage KOA group, and patients in stages 3-4 were included in the late-stage KOA group. In cases where different staging was detected between the right and left knees, the knee with the more advanced radiological stage was considered to provide the most clinically meaningful approach in comparative analyses, and evaluations were performed unilaterally accordingly.

### Insall–Salvati Ratio Evaluation

Lateral knee radiographs of participants, taken in a standing weight-bearing position with knee flexion at approximately 30°, were examined via the hospital Picture Archiving and Communication System (PACS). The ISR was calculated as the ratio of PTL to DLP. Measurements were made with digital calibration with an accuracy of 0.01 mm. The normal ISR value is approximately 1; ISR ≤ 0.8 is consistent with patella baja, ≥1.2 with patella alta.^26^ Although these thresholds are useful for clinical description, ISR was treated as a continuous (dimensionless) ratio in the primary analyses because values outside these cutoffs were infrequent in this cohort, limiting the interpretability of categorical subgroup comparisons.

Transverse-plane PF parameters such as the patellar tilt angle or Patella Index were not assessed because their quantification requires standardized axial (skyline/Merchant) radiographs and/or MRI. These modalities were not included in the imaging protocol, and no additional imaging was obtained solely for research purposes to avoid extra radiation exposure from additional axial radiographs and to maintain feasibility by not requesting MRI.

### Ultrasonographic Femoral Cartilage Measurement

The FCT was measured bilaterally using a high-resolution linear ultrasound probe (7-12 MHz; Siemens, Germany). Measurements were taken at 3 anatomical points (medial femoral condyle, intercondylar space, and lateral femoral condyle) through the suprapatellar window with participants in the supine position and the knee at maximum flexion.^15^ Right and left-knee cartilage thicknesses were calculated by averaging the measurements obtained from the 3 points for each knee. All measurements were performed by 2 independent observers with at least 5 years of experience in musculoskeletal US. The measurement protocol focused specifically on FCT to test the cartilage–ISR relationship, which is the hypothesis of the study; other US findings such as osteophytes or meniscus extrusion were not systematically quantitatively graded in this protocol. Inter-observer agreement was evaluated using the intraclass correlation coefficient (ICC), and ICC = 0.92 (95% CI: 0.87-0.96). This value indicates an excellent level of measurement reliability among observers.

### Statistical Analysis

Statistical analyses were performed using IBM SPSS Statistics for Windows, version 22.0 (IBM SPSS Corp.; Armonk, NY, USA). The distribution of continuous variables was assessed using skewness and kurtosis; values within ±2 were considered indicative of approximate normality (George & Mallery).^27^ Although normality was rejected for some variables by the Shapiro–Wilk and Kolmogorov–Smirnov tests, parametric methods were retained given the equal group sizes (n = 52 per group) and the robustness of these methods to modest departures from normality when distributions are approximately symmetric. Between-group comparisons were performed using independent-samples t tests for continuous variables and chi-square tests for categorical variables. Associations between continuous variables were examined using Pearson correlation. Because multiple pairwise correlations were tested within each stage-specific correlation matrix, *P*-values were adjusted using the Benjamini–Hochberg false discovery rate (FDR) procedure and are reported as q-values (q < 0.05 considered statistically significant for these exploratory correlations). To control for potential confounding, analysis of covariance (ANCOVA) was performed where appropriate, with covariates including age and BMI. The ISR was analyzed as a continuous measure in the primary analyses; although clinical cutoffs exist for patella baja/alta (ISR ≤0.8 or ≥1.2), values outside these thresholds were scarce in this cohort, and threshold-based subgroup analyses were therefore not emphasized as primary analyses. Receiver operating characteristic (ROC) curve analysis was performed to evaluate the ability of mean FCT to discriminate late-stage from early-stage KOA. Additionally, multivariable linear regression analyses (enter method) were conducted to identify independent factors associated with mean FCT (mm) and mean ISR. For the FCT model, KOA stage (1 = early, 2 = late), mean ISR, age (years), sex (1 = female, 2 = male), and BMI (kg/m^2^) were entered simultaneously. For the ISR model, KOA stage, age, sex, and BMI were entered. Unstandardized coefficients (B) with 95% CIs were reported. Model assumptions were evaluated using residual diagnostics, and multicollinearity was assessed using tolerance and variance inflation factors (VIF); VIF values ranged from 1.036 to 1.469 for the FCT model and from 1.067 to 1.447 for the ISR model. The area under the curve (AUC) with 95% CIs was reported, and the optimal cutoff was determined using the Youden index; this cutoff was internally derived from the study sample and should be interpreted as exploratory. All tests were two-sided. For correlation analyses, q < 0.05 was considered statistically significant; for all other analyses, *P* < .05 was considered statistically significant.

## Results

A total of 104 patients were included in the study, divided into early-stage KOA (n = 52) and late-stage KOA (n = 52). When the gender distribution of the participating patients was examined, the proportion of women was 76.9% (n = 40) and the proportion of men was 23.1% (n = 12) in the early-stage KOA group; while in the late-stage KOA group, the proportion of women was 88.5% (n = 46) and the proportion of men was 11.5% (n = 6). No statistically significant difference was found between the groups in terms of gender distribution (*χ*^2 ^= 2.419; *P* = .120).

The demographic and clinical characteristics of the groups are presented in Table 1. The mean age (62.63 ± 6.44 years) and BMI values ​​(30.93 ± 2.95 kg/m^2^) of patients in the late-stage KOA group were found to be statistically significantly higher than those in the early-stage group (*P* < .001 and *P* = .013, respectively).

When clinical and functional parameters were examined, it was found that walking time, VAS pain score, and WOMAC (Pain, Stiffness, Function, and Total) scores were significantly higher in the late-stage KOA group compared to the early-stage group; however, the total KOOS score was significantly lower (*P* < .001). In terms of structural parameters, FCT was found to be significantly thinner in the late-stage group (1.76 ± 0.25 mm) compared to the early-stage group (2.16 ± 0.31 mm) (*P* < .001). However, no statistically significant difference was observed between the groups in terms of the mean ISR (*P* = .554).

The ANCOVA analysis (Table 2), conducted to control for potential confounding effects of age and BMI differences between groups, showed that the differences between groups were maintained even after adjusting for age and BMI. Accordingly, the differences between groups in walking time, FCT, VAS, KOOS, and WOMAC scores remained statistically significant, independent of age and BMI (*P* < .001). In the adjusted analysis, no significant difference was found between stages in terms of ISR values ​​(*P* = .419).

In an exploratory side-specific evaluation (Table 3), FCT was significantly lower in both the right and left knees in late-stage disease patients (*P* < .001). When ISR values ​​were examined, no difference was observed between stages in the left knee (*P* = .096), while in the right knee, the ISR values ​​of late-stage patients (1.01 ± 0.12) were statistically significantly lower than those of early-stage patients (1.06 ± 0.16) (*P* = .039). This side-specific finding was considered exploratory and should be interpreted with caution.

### Correlation Analyses and the Relationship Between **Insall–Salvati Ratio** and Femoral Cartilage

Pearson correlation analyses were performed separately in the early- and late-stage KOA groups, and *P*-values were adjusted for multiple comparisons using the Benjamini–Hochberg FDR procedure (reported as q-values) (Tables 4 and 5).

In the early-stage KOA group (Table 4), the primary hypothesis regarding the relationship between mean ISR and mean FCT showed a moderate positive correlation (*r* = 0.331); however, this association did not remain statistically significant after FDR correction (q = 0.058). In this group, walking time correlated positively with age (*r* = 0.509, q < 0.001) and with symptom burden (VAS: *r* = 0.519, q < 0.001; WOMAC total: *r* = 0.519, q < 0.001) and correlated inversely with KOOS total (*r* = −0.394, q = 0.015). Pain severity (VAS) demonstrated strong correlations with patient-reported outcomes (KOOS total: *r* = −0.721, q < 0.001; WOMAC total: *r* = 0.810, q < 0.001), and KOOS total was strongly inversely correlated with WOMAC total (*r* = −0.913, q < 0.001).

In the late-stage KOA group (Table 5), the association between mean ISR and mean FCT was stronger and statistically significant after FDR adjustment (*r* = 0.527, q < 0.001), indicating that lower ISR values tended to co-occur with lower FCT in this cross-sectional sample. In addition, lower FCT correlated with longer walking time (*r* = −0.430, q = 0.004), higher pain intensity (VAS: *r* = −0.601, q < 0.001), and higher WOMAC total (*r* = −0.477, q < 0.001), while showing a positive correlation with KOOS total (*r* = 0.472, q < 0.001). Mean ISR also showed an inverse correlation with VAS (*r* = −0.349, q = 0.026). Walking time correlated with VAS (*r* = 0.570, q < 0.001), KOOS total (*r* = −0.540, q < 0.001), and WOMAC total (*r* = 0.532, q < 0.001), and KOOS total remained strongly inversely correlated with WOMAC total (*r* = −0.953, q < 0.001). A modest positive correlation was observed between BMI and VAS (*r* = 0.325, q = 0.040).

Overall, these findings describe cross-sectional co-variation between structural and clinical measures and should not be interpreted as evidence of causality.

In multivariable linear regression analyses, KOA stage (late vs. early) was independently associated with lower mean FCT (Table 6). Mean ISR also showed an independent positive association with mean FCT, whereas age, sex, and BMI did not show evidence of independent associations in this model (Table 6). The model explained a moderate proportion of variance in mean FCT (*R*^2 ^= 0.463; adjusted *R*^2 ^= 0.435; overall model *P* < .001). In contrast, the multivariable model for mean ISR did not show evidence of overall significance and explained little variance (*R*^2 ^= 0.035; adjusted *R*^2 ^= −0.004; overall model *P* = .472), and KOA stage was not independently associated with mean ISR (Table 6).

**Figure 1.**
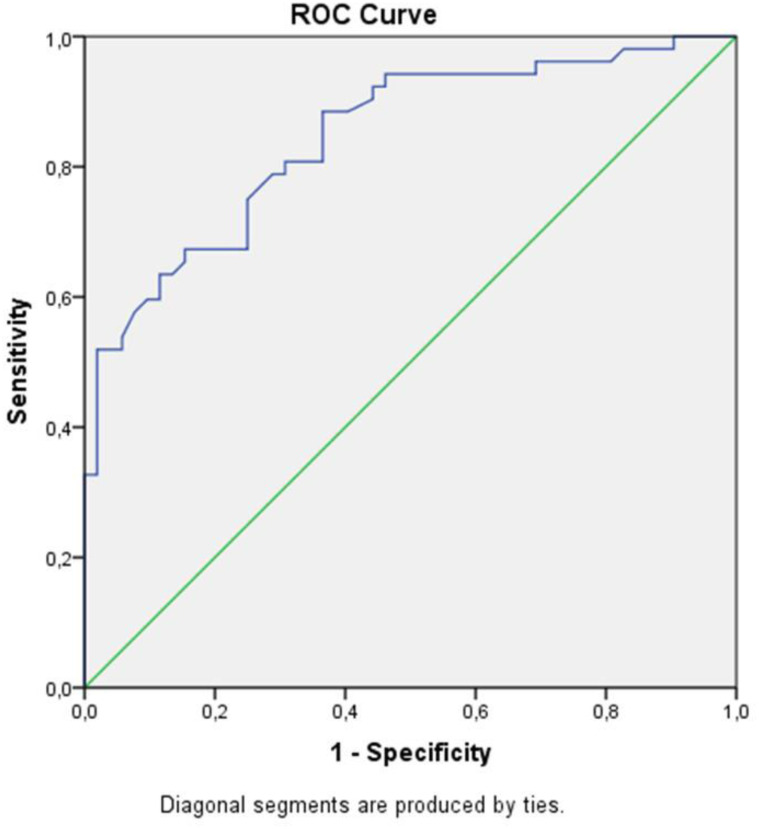
The ROC curve showing the ability of mean FCT to discriminate between early- and late-stage KOA. AUC = 0.845 (95% CI: 0.771-0.919; *P* < .001); cutoff ≤ 1.82 mm (Youden index) yielded 63.5% sensitivity and 88.5% specificity.

The ROC curve analysis suggested that mean FCT may have value for differentiating late-stage from early-stage KOA within this dataset (AUC = 0.845, 95% CI 0.771-0.919; *P* < .001) (Figure 1). A data-driven cutoff of ≤1.82 mm (maximizing the Youden index) corresponded to 63.5% sensitivity and 88.5% specificity for late-stage KOA (Figure 1). These discrimination estimates and the derived cutoff should be interpreted cautiously, given the cross-sectional design and the lack of external validation.

## Discussion

In this cross-sectional KOA cohort, ultrasonographic FCT was significantly lower in late-stage (K–L 3-4) than early-stage (K–L 1-2) disease, and this difference remained significant after adjustment for age and BMI. By contrast, mean ISR did not differ between stages in the adjusted analysis. In the exploratory side-specific comparison, FCT was reduced in both knees in late-stage KOA, whereas the small right-knee ISR difference should be interpreted cautiously because laterality-related biomechanical factors (e.g., limb dominance) were not assessed; therefore, definitive laterality inferences cannot be drawn.

Age and obesity are well-established risk factors for KOA and contribute to disease onset and progression through both mechanical loading and metabolic/inflammatory pathways.^28^^,^^29^ In a meta-analysis of prospective studies, overweight and obesity were associated with approximately 2.5-fold and 4.6-fold higher risks of knee OA compared with normal weight.^30^ In this cohort, the late-stage group was older and had a higher mean BMI, which is consistent with this risk factor profile.

In the study by Bozan et al,^^31^^ the 10-meter walking time was found to be significantly longer in patients with OA compared to the control group. In this study, the 100-meter walking time was significantly higher in patients with late-stage KOA compared to patients with early-stage KOA. These findings suggest that greater radiographic disease severity is associated with poorer walking performance, indicating a potential decline in functional capacity with advancing KOA.

Evidence on patellar height (ISR) and KOA severity is mixed. Wang et al^^32^^ assessed sagittal patellar alignment using the modified ISR and reported that patella alta was significantly associated with KOA after adjustment for covariates. Tanamas et al^^33^^ reported that PF geometry indices were associated with patellar cartilage volume. By contrast, Khoroushi et al^^34^^ reported that, in their correlation analysis, the association between the ISR and increasing MRI cartilage injury grade was weak in participants aged <50 years (rho ≈ 0.21) and negligible in those aged >50 years (rho ≈ 0.03).^34^ Recent imaging work suggests that patellar height measures and other PF alignment/morphology parameters may relate to radiographic KOA severity and that select PF morphology measures (e.g., the Tibial Tubercle–Trochlear Groove distance) may also be associated with lateral TF structural abnormalities, underscoring the value of multi-parameter PF assessment beyond sagittal ISR alone.^8^^,^^13^ In this study, ISR was not stage-discriminative after adjustment, suggesting that sagittal patellar height alone may be insufficient as an independent marker of radiographic severity in primary TF KOA.

Although ISR did not differ by stage, within-stage analyses showed that ISR correlated with mean FCT in late-stage KOA (*r* = 0.527, q < 0.001) but not after correction in early-stage KOA. This pattern may indicate that co-variation between patellar height and TF cartilage morphology becomes more detectable in advanced disease; however, causal interpretations are not possible in a cross-sectional design, and the association may reflect shared unmeasured biomechanical factors (e.g., alignment, muscle strength, meniscal pathology). In multivariable regression, KOA stage and ISR were independently associated with mean FCT, whereas KOA stage was not independently associated with ISR, further supporting that ISR should not be used as a stand-alone severity marker.

Ultrasonography-based FCT measurement provides a direct quantitative assessment of FC morphology and has shown acceptable validity for assessing knee cartilage thickness and degenerative changes when compared with reference imaging modalities.^14^,^15^^-^^16^ This methodological distinction may partly explain why FCT differentiated radiographic stage in the current sample, while radiography detects cartilage loss indirectly via joint-space narrowing. Consistent with prior studies, ultrasound cartilage findings indicative of greater structural degeneration were associated with higher pain severity and worse WOMAC scores, supporting the potential value of US as an accessible adjunct for structural assessment.^35^^,^^36^

Mean FCT demonstrated good discrimination of late-stage KOA (AUC = 0.845). The internally derived threshold (≤1.82 mm) showed high specificity but moderate sensitivity and therefore requires external validation before clinical application. Clinically, these findings support the potential role of ultrasonographic FCT as an accessible adjunct to radiographs for structural stratification and may help identify patients who could benefit from earlier load-modifying rehabilitation. While ISR was not stage-discriminative in this cohort, marked patellar height abnormalities (e.g., patella alta) may still identify patients in whom early PF load–modifying rehabilitation (e.g., hip-knee exercise, taping/bracing, and foot orthoses) is clinically justifiable, with the goal of reducing PF joint stress and potentially mitigating further structural burden.^10^^,^^37^ However, the cartilage-sparing effects of such interventions in KOA remain uncertain, and the data should not be interpreted as evidence of proven chondroprotection.

As expected, late-stage patients reported worse pain, function, and walking performance. Prior studies have reported relationships between K–L grade, ultrasonographic structural findings, and symptom/function scores.^35^^,^^36^ Slower timed-walk performance has also been reported in OA cohorts.^31^ In end-stage KOA, substantial impairments across KOOS subscales have been reported, with knee-related quality of life typically among the most affected domains.^38^ These findings extend these data by showing that US-measured FCT is aligned with both radiographic stage and patient-reported outcomes in this cohort.

This study has several strengths. The most important factor enhancing the methodological strength of this study is its direct evaluation of the association between patellar height (ISR) and TF cartilage using US, a non-invasive and cost-effective method, a finding that is limited in the literature. The adoption of the “index knee” approach to prevent bias due to bilateral data repetition in statistical analysis, and the standardization of major risk factors such as age and BMI using ANCOVA analysis, significantly increased the internal validity of the results. Furthermore, the high inter-observer agreement supports the idea that US is a reproducible and reliable clinical alternative to MRI in this field.

Key limitations are the cross-sectional design; limited power for categorical ISR subgroups (few patella baja cases); exclusion of isolated PF OA (limiting generalizability); assessment of patellar alignment only by sagittal ISR without axial PF metrics; and lack of other important biomechanical confounders (alignment, quadriceps strength, meniscal pathology, activity level). Ultrasonography assessment was limited to FCT and did not systematically grade osteophytes or meniscal extrusion, and MRI was not available as a reference method. Side-specific comparisons were exploratory, and limb dominance was not recorded.

In conclusion, ultrasonographic FCT differentiated early and late radiographic KOA and was consistently associated with pain, functional scores, and walking performance after adjustment for age and BMI. Mean ISR did not differ between stages after adjustment and should not be considered an independent marker of radiographic severity. The within-stage ISR–FCT associations should be viewed as hypothesis-generating and warrant confirmation in prospective, longitudinal studies incorporating key biomechanical confounders.

### Data Availability Statement:

The data that support the findings of this study are available on request from the corresponding author.

### Artificial Intelligence Usage Statement:

This study used DeepL to improve language and edit grammar during the preparation of the manuscript.

### Ethics Committee Approval:

Ethical committee approval was received from the Ethics Committee of University of Fırat (Approval No.: 2024/07-31; Date: May 9, 2024).

### Informed Consent:

Written informed consent was obtained from the patients who agreed to take part in the study.

### Peer-review:

Externally peer-reviewed.

### Author Contributions:

Concept – M.Ş.E.; Design – M.Ş.E., M.K.; Supervision – M.Ş.E., M.K.; Resources – N.Y.; Materials – N.Y.; Data Collection and/or Processing – N.Y.; Analysis and/or Interpretation – M.Ş.E.; Literature Search – N.Y.; Writing – M.K, N.Y.; Critical Review – M.Ş.E., M.K.

### Declaration of Interests:

The authors have no conflicts of interest to declare.

### Funding:

The authors declare that this study received no financial support.

## Figures and Tables

**Table 1.** Comparison of Clinical and Functional Parameters in Early and Late-Stage Knee Osteoarthritis Patients

**Parameter**	**Early Stage (n = 52)** **(Mean ± SD)**	**Late Stage (n = 52)** **(Mean ± SD)**	***t***	***P***
Age (years)	55.12 ± 6.63	62.63 ± 6.44	−5.870	**<.001**
BMI (kg/m^2^)	29.52 ± 2.73	30.93 ± 2.95	−2.520	**.013**
Walking time (secs)	108.27 ± 11.18	142.90 ± 20.90	−10.540	**<.001**
ISR (Index knee)	1.06 ± 0.15	1.05 ± 0.13	0.590	.554
FCT (mm) (Index knee)	2.16 ± 0.31	1.76 ± 0.25	7.350	**<.001**
VAS	3.83 ± 1.38	6.62 ± 1.57	−9.600	**<.001**
KOOS total	73.02 ± 7.03	54.47 ± 7.94	12.608	**<.001**
WOMAC pain	7.94 ± 3.63	14.17 ± 2.81	−9.790	**<.001**
WOMAC stiffness	3.37 ± 1.98	6.10 ± 1.66	−7.620	**<.001**
WOMAC function	31.52 ± 10.08	54.08 ± 6.14	−13.780	**<.001**
WOMAC total	42.88 ± 14.33	73.73 ± 9.46	−12.952	**<.001**

BMI, body mass index; FCT, femoral cartilage thickness; ISR, Insall–Salvati ratio; KOA, knee osteoarthritis; KOOS, Knee Injury and Osteoarthritis Outcome Score; VAS, Visual Analog Scale; WOMAC, Western Ontario and McMaster Universities Osteoarthritis Index. Bold values indicate statistically significant differences between groups (*P* < 0.05).

**Table 2.** Comparison of Clinical Parameters Between Knee Osteoarthritis Stages with ANCOVA

**Parameter**	**Early Stage** **(Mean ± SD)**	**Late Stage (Mean ± SD)**	***F* (*df*)**	P*	**Partial Eta Squared (η^2^)**
Walking Time (secs)	110.99 ± 2.44	140.19 ± 2.44	62.475 (1,100)	**<.001**	0.385
ISR (Index knee)	1.064 ± 0.15	1.048 ± 0.13	0.659 (1,100)	.419	0.007
FCT (Index knee) (mm)	2.16 ± 0.31	1.76 ± 0.25	46.699 (1,100)	**<.001**	0.318
VAS	3.99 ± 1.38	6.45 ± 1.57	56.632 (1,100)	**<.001**	0.362
KOOS Total	73.02 ± 7.03	54.47 ± 7.94	104.195 (1,100)	**<.001**	0.510
WOMAC Total	43.94 ± 1.82	72.67 ± 1.82	108.413 (1,100)	**<.001**	0.520

FCT, femoral cartilage thickness; ISR, Insall–Salvati ratio; KOA, knee osteoarthritis; KOOS, Knee Injury and Osteoarthritis Outcome Score; VAS, Visual Analog Scale; WOMAC, Western Ontario and McMaster Universities Osteoarthritis Index.

*ANCOVA. Bold values indicate statistically significant results after adjustment for age and BMI using ANCOVA (*P* < .05).

**Table 3.** Comparison of Right- and Left-Knee Insall–Salvati Ratio and Femoral Cartilage Thickness in Early- and Late-Stage Knee Osteoarthritis Patients

**Parameter**	**Early Stage (n = 52) (Mean ± SD)**	**Late Stage (n = 52) (Mean ± SD)**	***t***	***P***
FCT Left (mm)	2.12 ± 0.34	1.68 ± 0.24	6.66	**<.001**
ISR Left	1.09 ± 0.16	1.04 ± 0.14	1.68	.096
FCT Right (mm)	2.14 ± 0.29	1.68 ± 0.22	8.84	**<.001**
ISR Right	1.06 ± 0.16	1.01 ± 0.12	2.09	**.039**

FCT, femoral cartilage thickness; ISR, Insall–Salvati ratio; KOA, knee osteoarthritis. Bold values indicate statistically significant differences between groups (p < 0.05).

**Table 4.** Pearson Correlations Between Clinical Variables in Individuals with Early-Stage Knee Osteoarthritis

​	**Age (Years)**	**BMI**	**Walking Time**	**ISR (Index Knee)**	**FCT (Index Knee)**	**VAS**	**KOOS Total**	**WOMAC Total**
Age	1	0.274 q = 0.126	0.509 q **≤** **0.001**	0.080 q = 0.652	0.138 q = 0.512	0.193 q = 0.298	−0.242 q = 0.170	0.241 q = 0.170
BMI	0.274 q = 0.126	1	0.257 q = 0.154	−0.078 q = 0.652	0.117 q = 0.572	0.180 q = 0.332	−0.091 q = 0.652	0.119 q = 0.572
Walking Time	0.509 q** ≤ 0.001**	0.257 q = 0.154	1	0.065 q = 0.697	−0.015 q = 0.916	0.519 q **≤** **0.001**	−0.394 q = 0**q = 0.015**	0.519 q **≤** **0.001**
ISR(Index knee)	0.080 q = 0.652	−0.078 q = 0.652	0.065 q = 0.697	1	0.331 q = 0.058	−0.081 q = 0.652	−0.043 q = 0.790	0.079 q = 0.652
FCT(Index knee)	0.138 q = 0.512	0.117 q = 0.572	−0.015 q = 0.916	0.331 q = 0.058	1	−0.283 q = 0.118	0.232 q = 0.183	−0.318 q = 0.067
VAS	0.193 q = 0.298	0.180 q = 0.332	0.519 q **≤** **0.001**	−0.081 q = 0.652	−0.283 q = 0.118	1	−0.721 q **≤** **0.001**	0.810 q **≤** **0.001**
KOOS Total	−0.242 q = 0.170	−0.091 q = 0.652	−0.394 q = 0**q = 0.015**	−0.043 q = 0.790	0.232 q = 0.183	−0.721 q **≤** **0.001**	1	−0.913 q **≤** **0.001**
WOMAC Total	0.241 q = 0.170	0.119 q = 0.572	0.519 q **≤** **0.001**	0.079 q = 0.652	−0.318 q = 0.067	0.810 q **≤** **0.001**	−0.913 q **≤** **0.001**	1

q-values denote *P*-values adjusted using the Benjamini–Hochberg FDR procedure for multiple comparisons.

BMI, body mass index; FCT, femoral cartilage thickness; ISR, Insall–Salvati ratio; KOA, knee osteoarthritis; KOOS, Knee Injury and Osteoarthritis Outcome Score; VAS, Visual Analog Scale; WOMAC, Western Ontario and McMaster Universities Osteoarthritis Index.

**Table 5.** Pearson Correlations Between Clinical Variables in Individuals with Late-Stage Knee Osteoarthritis

​	**Age (Years)**	**BMI**	**Walking Time**	**ISR (Index Knee)**	**FCT (Index Knee)**	**VAS**	**KOOS Total**	**WOMAC Total**
Age	1.000 –	0.254 q = 0.121	0.148 q = 0.393	0.087 q = 0.630	0.075 q = 0.669	0.114 q = 0.513	−0.002 q = 0.989	−0.007 q = 0.989
BMI	0.254 q = 0.121	1.000 −	0.142 q = 0.401	−0.160 q = 0.388	−0.158 q = 0.388	0.325**q = 0.040**	−0.150 q = 0.393	0.060 q = 0.724
Walking Time	0.148 q = 0.393	0.142 q = 0.401	1.000 −	−0.216 q = 0.204	−0.430**q = 0.004**	0.570 q** ≤ 0.001**	−0.540 q** ≤ 0.001**	0.532 q **≤** **0.001**
ISR (Index knee)	0.087 q = 0.630	−0.160 q = 0.388	−0.216 q = 0.204	1.000 −	0.527 q** ≤ 0.001**	−0.349**q = 0.026**	0.290 q = 0.074	−0.262 q = 0.113
FCT (Index knee)	0.075 q = 0.669	−0.158 q = 0.388	−0.430**q = 0.004**	0.527 q** ≤ 0.001**	1.000 −	−0.601 q **≤** **0.001**	0.472 q ≤ **0.001**	−0.477 q **≤** **0.001**
VAS	0.114 q = 0.513	0.325**q = 0.040**	0.570 q **≤** **0.001**	−0.349**q = 0.026**	−0.601 q **≤** **0.001**	1.000 −	−0.565 q **≤** **0.001**	0.545 q **≤** **0.001**
KOOS Total	−0.002 q = 0.989	−0.150 q = 0.393	−0.540 q **≤** **0.001**	0.290 q = 0.074	0.472 q **≤** **0.001**	−0.565 q **≤** **0.001**	1.000 −	−0.953 q **≤** **0.001**
WOMAC Total	−0.007 q = 0.989	0.060 q = 0.724	0.532 q **≤** **0.001**	−0.262 q = 0.113	−0.477 q** ≤ 0.001**	0.545 q **≤** **0.001**	−0.953 q **≤** **0.001**	1.000 −

q-values denote *P*-values adjusted using the Benjamini–Hochberg FDR procedure for multiple comparisons.

BMI, body mass index; FCT, femoral cartilage thickness; ISR, Insall–Salvati ratio; KOA, knee osteoarthritis; KOOS, Knee Injury and Osteoarthritis Outcome Score; VAS, Visual Analog Scale; WOMAC, Western Ontario and McMaster Universities Osteoarthritis Index.

**Table 6.** Multivariable Linear Regression Models for Mean Femoral Cartilage Thickness and Mean Insall–Salvati Ratio

**Predictor**	**Model 1: Mean FCT (mm) *B* (95% CI)**	***P***	**Model 2: Mean ISR *B* (95% CI)**	***P***
KOA stage (Late vs. Early) (Group stage: 2 vs. 1)	−0.411 (−0.530, −0.293)	<.001	−0.028 (−0.091, 0.035)	.380
Mean ISR (per 1-unit increase)	0.829 (0.457, 1.202)	<.001	–	–
Age (years)	0.003 (−0.005, 0.011)	.492	0.003 (−0.002, 0.007)	.217
Sex (Male vs. Female) (Sex: 2 vs. 1)	0.041 (−0.097, 0.179)	.556	−0.022 (−0.096, 0.051)	.549
BMI (kg/m^2^)	0.003 (−0.016, 0.022)	.755	−0.008 (−0.018, 0.002)	.124

Values are unstandardized coefficients (B) with 95% CIs.

Model fit (Model 1, Mean FCT): *R*^2 ^= 0.463; Adjusted *R*^2 ^= 0.435; *F* (5,98) = 16.883; *P* < .001.

Model fit (Model 2, Mean ISR): *R*^2 ^= 0.035; Adjusted *R*^2 ^= −0.004; *F* (4,99) = 0.891; *P* = .472.

KOA:Knee Osteoarthritis, BMI:Body Mass Index, FCT:Femoral Cartilage Thickness, ISR:Insall–Salvati Ratio

BMI, body mass index; FCT, femoral cartilage thickness; ISR, Insall–Salvati ratio; KOA, knee osteoarthritis.
